# The Stress of Measuring Plantar Tissue Stress in People with Diabetes-Related Foot Ulcers: Biomechanical and Feasibility Findings from Two Prospective Cohort Studies

**DOI:** 10.3390/s24082411

**Published:** 2024-04-10

**Authors:** Chantal M. Hulshof, Madelyn Page, Sjef G. van Baal, Sicco A. Bus, Malindu E. Fernando, Lisette van Gemert-Pijnen, Kilian D. R. Kappert, Scott Lucadou-Wells, Bijan Najafi, Jaap J. van Netten, Peter A. Lazzarini

**Affiliations:** 1 Amsterdam UMC Location University of Amsterdam, Rehabilitation Medicine, Meibergdreef 9, 1105 AZ Amsterdam, The Netherlands; 2 Amsterdam Movement Sciences, Ageing & Vitality and Rehabilitation & Development, 1081 BT Amsterdam, The Netherlands; 3 School of Public Health and Social Work, Queensland University of Technology, Brisbane, QLD 4059, Australia; 4 ZGT Academy, ZGT, 7600 SZ Almelo, The Netherlands; 5 Southwestern Academic Limb Salvage Alliance (SALSA), Department of Surgery, Keck School of Medicine of University of Southern California, Los Angeles, CA 90033, USA; 6 Rancho Los Amigos National Rehabilitation Center, Los Angeles, CA 90242, USA; 7 Ulcer and Wound Healing Consortium (UHEAL), Queensland Research Centre for Peripheral Vascular Disease, College of Medicine and Dentistry, James Cook University, Townsville, QLD 4811, Australia; 8 Department of Vascular and Endovascular Surgery, John Hunter Hospital, New Lambton Heights, Newcastle, NSW 2305, Australia; 9 Department of Psychology, Health & Technology, University of Twente, 7522 NB Enschede, The Netherlands; 10 Department of Surgery, ZGT, 7600 SZ Almelo, The Netherlands; 11 Allied Health Research Collaborative, Metro North Hospital and Health Service, Brisbane, QLD 4032, Australia; 12 Interdisciplinary Consortium on Advanced Motion Performance, Baylor College of Medicine, Houston, TX 77030, USA

**Keywords:** offloading, plantar pressure, shear stress, daily-life activity, adherence, diabetic foot, foot ulcer

## Abstract

Reducing high mechanical stress is imperative to heal diabetes-related foot ulcers. We explored the association of cumulative plantar tissue stress (CPTS) and plantar foot ulcer healing, and the feasibility of measuring CPTS, in two prospective cohort studies (Australia (AU) and The Netherlands (NL)). Both studies used multiple sensors to measure factors to determine CPTS: plantar pressures, weight-bearing activities, and adherence to offloading treatments, with thermal stress response also measured to estimate shear stress in the AU-study. The primary outcome was ulcer healing at 12 weeks. Twenty-five participants were recruited: 13 in the AU-study and 12 in the NL-study. CPTS data were complete for five participants (38%) at baseline and one (8%) during follow-up in the AU-study, and one (8%) at baseline and zero (0%) during follow-up in the NL-study. Reasons for low completion at baseline were technical issues (AU-study: 31%, NL-study: 50%), non-adherent participants (15% and 8%) or combinations (15% and 33%); and at follow-up refusal of participants (62% and 25%). These underpowered findings showed that CPTS was non-significantly lower in people who healed compared with non-healed people (457 [117; 727], 679 [312; 1327] MPa·s/day). Current feasibility of CPTS seems low, given technical challenges and non-adherence, which may reflect the burden of treating diabetes-related foot ulcers.

## 1. Introduction

### 1.1. Global Burden of Diabetes and Foot Ulcers

Diabetes is the most rapidly growing cause of the global disease burden [1], affecting an estimated 537 million people worldwide [2,3]. Diabetes-related foot ulcers are the leading cause of the global diabetes-related hospitalisations, amputations, and disease burden, affecting an estimated 20 million people yearly, with at least another 130 million people at risk [4,5]. These ulcers substantially reduce quality of life and impose a large disease burden on patients and society [5,6,7,8]. Unfortunately, not all factors in the ulcer healing process are completely understood. For instance, the exact role that different mechanical stresses play on plantar tissue in relation to ulcer healing in removable offloading treatments (i.e., devices or footwear) is not completely understood. Currently, this has only been objectively investigated to a limited extent with sensor devices [9]. To reduce this large burden on patients and society, a better understanding of the healing mechanisms involved in diabetes-related foot ulcers is crucial [10].

### 1.2. Mechanical Stress Is Still Poorly Understood

People with diabetes-related foot ulcers often have high mechanical stresses at the ulcer site that originally caused the ulcer and subsequently delay its healing [6,11]. Offloading the ulcer site to reduce these high mechanical stresses is a cornerstone of treatment to heal foot ulcers [6,12,13]. Plantar pressure is one factor that contributes to mechanical stress that has been known, measured, and investigated for many years. Plantar pressure has been found to be significantly reduced at the ulcer site when using knee-high removable devices compared to ankle-high removable devices [14]. Interestingly, despite this difference in plantar pressure between these removable devices, studies have found no difference in healing outcomes [14]. To better understand why this is the case, we likely need to consider other factors that also contribute to the overall mechanical stresses affecting the ulcer site. This is especially important because removable offloading devices remain the treatment most frequently used in clinical practice [15,16]. Overall mechanical stress is the result of all the vertical (i.e., plantar pressure) and horizontal (i.e., shear) stresses acting on the foot during weight-bearing activities [9]. Additionally, mechanical stress is also dependent on the frequencies of these weight-bearing activities and whether or not an offloading device is used during such activities (i.e., adherence) [9]. Combined, those factors are thought to determine the overall mechanical stress, or “cumulative plantar tissue stress” (CPTS), on a location of the foot, such as the ulcer site [9]. This CPTS concept was first introduced in 2003 by Maluf and Mueller [17], yet only one pilot study has since explored the association of CPTS with diabetes-related foot ulcer healing [18]. This lack of research is somewhat surprising, considering international guidelines that have continually recommended pursuing more studies on CPTS [19]. Therefore, our understanding of the influence of overall mechanical stresses on diabetes-related foot ulcer healing remains poor, and more research is urgently needed [9,19].

### 1.3. Challenging Equipment of Sensors

A potential explanation for this paucity of research may lie in the sensor equipment required to objectively measure and determine CPTS. There is no single, inexpensive, easy-to-use, and validated sensor-based equipment available to continuously measure all underlying factors and in turn determine CPTS [9]. Thus, each individual factor currently needs to first be measured separately using different sensor devices and software packages. Then, the collected data from each individual factor need to be combined using complex algorithms to eventually determine CPTS [9]. Employing multiple sensor devices with different usability results in different data formats, introducing challenges in the integration of data and the determination of CPTS. Therefore, in addition to more studies being needed to understand the association between CPTS and ulcer healing, insight into the feasibility of measuring CPTS in people with diabetes-related foot ulcers is also needed.

### 1.4. Aims and Hypotheses

Thus, the aims of this study were as follows: (i) to investigate the association between CPTS with ulcer healing in people with diabetes-related plantar foot ulcers using removable offloading treatments and (ii) to explore the feasibility of measuring CPTS. To investigate this, we combined data from two prospective observational cohort studies that were conducted with similar protocols. Our hypothesis was that ulcer healing is associated with lower CPTS, and that measuring CPTS is feasible in people with diabetes-related foot ulcers.

## 2. Materials and Methods

### 2.1. Design and Participants

Two 12-week prospective observational cohort studies investigating CPTS in people with a plantar diabetes-related foot ulcer were used to address the study aims: the *Towards an Objective Plantar Stress threshold for Diabetic Foot Ulcer healing study* from Australia (“Australian [AU] study”) and the *Comprehensive load-capacity model of diabetic foot ulcer healing study* from the Netherlands (“Netherlands [NL] study”). The recruitment period of the AU-study was from April 2019 until June 2020 and for the NL-study, from March 2020 until December 2022. These ‘sister studies’ were led by two principal investigators (J.J.v.N. and P.A.L.) who oversaw both study protocols, with both study protocols being as similar as possible, taking into account local equipment availability (see Section 2.3). All study procedures were conducted in accordance with the Declaration of Helsinki, and written informed consent was obtained from all participants prior to inclusion.

In the AU-study, inclusion criteria were ambulant adults with type 2 diabetes mellitus, peripheral neuropathy (using a 10-g monofilament and tuning fork [6]), plantar diabetes-related foot ulcer (i.e., full thickness ulcer on the plantar surface of the foot [20]), and the cognitive ability and willingness to cooperate with the study procedures. In participants with multiple ulcers, the index ulcer, defined as the largest ulcer in surface area, was investigated [21]. Exclusion criteria were treatment with a total contact cast, severe peripheral artery disease (toe pressure < 30 mmHg [22]), moderate or severe foot infection [23], active Charcot neuro-osteoarthropathy or fall risk (>2 falls in past 12 months [24]). Participants were recruited from six diabetic foot clinics in Brisbane, Australia. The Human Research Ethics Committee of Metro North Health (Brisbane, Australia) approved the protocol for this study (registration number: 44098).

In the NL-study, inclusion criteria were ambulant adults with type 1 or type 2 diabetes mellitus, peripheral neuropathy (using a 10-g monofilament and tuning fork [6]), and plantar diabetes-related foot ulcer (i.e., full thickness ulcer on the plantar surface of the foot [20]). Exclusion criteria were treatment with a total contact cast, plantar ulcer on the contralateral foot, also having a non-plantar ulcer (on the contra- or ipsilateral foot), severe peripheral artery disease (toe pressure < 30 mmHg [22]), moderate or severe foot infection [23], active Charcot neuro-osteoarthropathy, or an open amputation wound. Participants were recruited from three hospitals in the Netherlands (Amsterdam UMC, ZGT and BovenIJ hospital). This study was registered in the Netherlands trial register (registration number: NL9117) and requirement for ethical review of this study was waived under the Medical Research Involving Human Subjects Act in the Netherlands by the medical ethics committee of Amsterdam UMC (registration number: W19_429#19.495). 

### 2.2. Participant Characteristics

In both studies, the following demographic and medical characteristics were collected at the baseline visit (0 weeks): age, sex, body mass index, diabetes duration, HbA1c, ulcer history, amputation history, peripheral artery disease, nephropathy, and retinopathy [21]. In addition, in the NL-study diabetes type was collected. Furthermore, in both studies the following ulcer characteristics were collected: duration of ulcer before study start, site, size, depth, University of Texas Wound Classification, signs of infection, and ischemia [20,21]. Additionally, the removable offloading device used for the treatment of the plantar ulcer was documented. 

### 2.3. Outcomes of Interest

The outcomes of interest for both studies were CPTS and its underlying factors, consisting of plantar pressure, daily weight-bearing activity, and adherence to using an offloading device plus, in the AU-study, plantar shear stress. See Table 1 for the overview and timing of the outcomes of interest measurements. In both studies, outcomes were measured in two conditions: the condition when the offloading device was used (i.e., in-device condition) and the condition when the offloading device was not used (i.e., non-device condition). In the AU-study, the in-device and non-device condition for the plantar pressure and shear stress measurements were randomly ordered by using computer generated randomisation software. In the NL-study, the order was not randomised.

#### 2.3.1. Plantar Pressure

In both studies, plantar pressures were measured in the in-device condition. To represent the non-device condition, plantar pressures were measured in the AU-study in the participant’s regular footwear and in the NL-study while walking barefoot. To protect the ulcer during the measurements in the non-device condition, a transparent film dressing was placed over the participants’ ulcer, and the number of steps in the non-device condition was kept to a minimum. Plantar pressures in the offloading device and in the regular footwear were measured during walking at a self-selected speed with the validated Pedar-X system at 50 Hz (Novel GmbH, Munich, Germany), using flexible insoles at the sock–insole interface with 99 capacitive sensors [25]. During the measurements, the time and distance were measured to calculate the walking speed, which was maintained constant between trials. At least 12 midgait steps from both feet were needed for a valid measurement [26]. Raw plantar pressure data were analysed using custom-made Matlab scripts (Mathworks, Natick, MA, USA, version R2022b) to calculate peak plantar pressure (PPP) and the pressure–time integral (PTI) at the ulcer site. Barefoot plantar pressures were measured using the validated EMED-X platform with 4 sensors/cm^2^ at 100 Hz (Novel GmbH, Munich, Germany), according to the validated 2-step protocol [27], repeated four times per foot with the average over four trials used. PPP and PTI were determined using the manufacturer’s software at the ulcer site.

#### 2.3.2. Daily Weight-Bearing Activity

In both studies, daily weight-bearing activities were measured with a validated tri-axial accelerometer (MoveMonitor, McRoberts, The Hague, The Netherlands) at 100 Hz within a range of −6 g to +6 g [28,29]. The accelerometer was worn at the L5 vertebrae for at least 6 days, except during water-related activities. At least 4 valid days with a minimum of 12 h of wearing time were required for the analysis [30,31]. A validated algorithm of McRoberts identified and determined the number of strides [32]. Daily weight-bearing activity was determined as the number of strides per day averaged over valid days.

#### 2.3.3. Adherence to Using an Offloading Device

In both studies, a temperature sensor (Orthotimer, Rollerwerk, Balingen, Germany) was placed in the device to measure the adherence to using the offloading device. This sensor measured absolute temperature every 15 min. The wearing time of the offloading device was determined from the temperature data using the Groningen algorithm, version 2 [33,34,35]. To determine adherence to using the offloading device, data on wearing time and the number of strides as measured with the tri-axial accelerometer (see Section 2.3.2. daily weight-bearing activity) were integrated. Next, the number of strides taken when using and not using the offloading device were determined. Adherence was then determined as the proportion of strides when wearing the offloading device of the total number of strides taken by the participant [36].

#### 2.3.4. Plantar Shear Stress

Given a lack of validated equipment to measure shear stress, in the AU-study plantar shear stress was estimated. For this, the thermal stress response, a surrogate outcome measure for plantar shear stress [37], was measured using the FLIR One infrared camera (FLIR Systems Inc., Wilsonville, OR, USA). In the NL-study, these thermal cameras were not available and hence this estimation of plantar shear stress was not measured. The thermal stress response was calculated by measuring plantar foot temperature pre- and post-walking for the in-device and non-device condition. A validated thermal stress response protocol was used whereby the pre-walk image was taken immediately preceding the walking trial and after five minutes of temperature acclimatization (i.e., non-weight-bearing). The post-walk image was taken immediately after walking [37]. For both images, participants were sitting on a treatment bench with their legs horizontal. The following equation was used to estimate the thermal stress response at the ulcer site and at the similar location on the contralateral foot (Equation (1)):

(1) $$Thermal\;stress\;response=\frac{\left(\frac{post-pre}{pre}\right)index\;foot}{\left(\frac{post-pre}{pre}\right)contralateral\;foot}$$

### 2.4. Cumulative Plantar Tissue Stress Model

CPTS per day was determined at the ulcer site by integrating data on plantar pressure, daily weight-bearing activity, adherence to using an offloading device, and thermal stress response, with the latter only included in the AU-study [9]. In both studies, CPTS was calculated according to CPTS model 1 (Equation (2)) [9].

(2) $$CPTS_{\mathrm{model}1}=\sum_{c=1}^{2}{\left(PTI_{\mathrm{ulcer}}\times \mathrm{number}\;\mathrm{of}\;\mathrm{strides}\right)}_{c}$$

Note: CPTS = cumulative plantar tissue stress, expressed in Mpa·s/day; c = condition of the foot: in offloading device (i.e., in-device) and without offloading device (i.e., non-device); PTI = pressure–time integral expressed in kPa·s at the ulcer location; number of steps expressed per day.

 

For the AU-study, CPTS was also calculated according to CPTS model 2 (Equation (3)), in which CPTS was weighted for plantar shear stress as estimated based on the thermal stress response.

(3) $$CPTS_{\mathrm{model}2}=\sum_{c=1}^{2}{\left(PTI_{\mathrm{ulcer}}\times \mathrm{number}\;\mathrm{of}\;\mathrm{strides}\times \mathrm{thermal}\;\mathrm{stress}\;\mathrm{response}\right)}_{c}$$

Note: CPTS = cumulative plantar tissue stress, expressed in MPa·s/day; c = condition of the foot: in offloading device (i.e., in-device) and without offloading device (i.e., non-device); PTI = pressure–time integral expressed in kPa·s at the ulcer location; number of steps expressed per day.

 

The mean CPTS per day was determined per valid day and subsequently averaged over all valid days.

### 2.5. Primary Outcomes

In both studies, the primary outcomes were healing of the index ulcer at the 12-week follow-up and ulcer reduction of ≥75% at 4 weeks. A healed ulcer was defined as follows: “intact skin, meaning complete epithelization without any drainage of the previous foot ulcer site” [20].

### 2.6. Feasibility Outcomes

To assess feasibility, the number of completed CPTS measurements was described, the reasons for non-completion were reported, and characteristics were compared between participants with complete CPTS data versus those without. We hypothesised that certain readily accessible patient characteristics may be associated with those that completed all CPTS measurements compared to those that did not, such as younger age, lower BMI, or decreased ulcer severity (size, depth, ischaemia, and duration). Such findings may mean that participants less likely to complete all CPTS measures could be identified in the future to enable more nuanced clinical discussions with these patients as to the benefits and harms of completing CPTS measures for clinical practice or research studies. We defined the following reasons for non-completion: “non-adherent participant”: a participant that did not adhere to the measurement protocol to obtain valid data for daily weight-bearing activity, one of the underlying CPTS factors; “technical issue”: a technical issue with the plantar pressure equipment, tri-axial accelerometer, temperature sensor device, or study laptop, either due to hardware/software issues or researcher error; “combination”: a combination of a non-adherent participant and technical issue; “participant refusal”: a participant that declined to participate in the (follow-up) measurements; and “drop-out”: a participant dropping-out for another reason than refusal (e.g., hospitalisation).

### 2.7. Sample Size Calculations

Both studies based the power calculations on an exploratory secondary analysis study of CPTS in people with diabetes-related plantar foot ulcers [18]. In the AU-study, ulcer healing was taken as the primary outcome for the power calculations, with a ~66% healing rate at 12 weeks assumed with best practice treatment [18]. A total of 60 participants were needed to detect a statistically significant difference in CPTS, with an estimated CPTS value of 140 (SD: 137) for healing and of 275 (SD: 209) for non-healing; alpha 0.05, beta 0.90, 2-tailed, and drop-out rate of 20% [18]. In the NL-study, ≥75% ulcer area reduction at 4 weeks was taken as the primary outcome, with an ulcer reduction of ≥75% at 4 weeks assumed with best practice treatment from ~70% of the participants [18]. A total of 40 participants were needed to detect a statistically significant difference in CPTS, with an estimated CPTS value of 140 (SD: 137) for a ≥75% reduction and 275 (SD: 209) for a <75% reduction; alpha 0.05, beta 0.80, 2-tailed, non-adherence rate of 20%, and drop-out rate of 10% [18].

### 2.8. Statistical Analysis

Baseline characteristics and all outcomes of interest were compared between people who healed versus people who did not heal at 12 weeks and between people who had and did not have CPTS data available using the Student’s *t*-test (normal distribution) and Mann–Whitney U test (non-normal distribution) for continuous variables and the Chi-square test for categorical variables. In addition, we determined the effect size (r = z-score Mann–Whitney U test/√number of measurements) for the outcomes of interest. The effect sizes for non-normally distributed values were interpreted as follows: ≥0.1 indicated a small effect, ≥0.3 a moderate effect, and ≥0.5 a large effect [38]. All statistical analysis were performed using SPSS Statistics for Windows, version 26 (IBM Corp., Armonk, NY, USA) with a significance level of α < 0.05.

## 3. Results

### 3.1. Participant Recruitment

**Figure 1:**
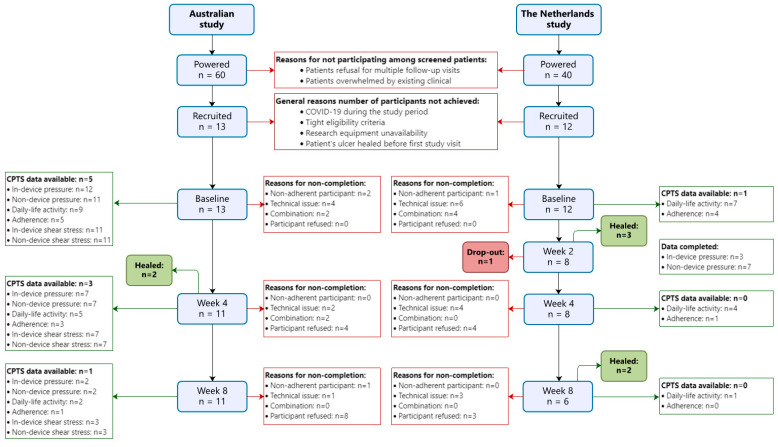
Flowchart of the number of participants, data availability for CPTS, and reasons for non-completion. See Section 2.6 for definitions on reasons.

Of the numbers required to recruit to reach adequate power according to our original sample size calculations, the AU-study recruited 13 (22%) of the required 60 participants and the NL-study recruited 12 participants (30%) of the required 40 participants. The primary reason for screened eligible patients not participating in the study was that they declined to consent to multiple follow-up study appointments, as they stated that they were already overwhelmed with their multiple existing clinical appointments. Otherwise, general reasons for not reaching the sample size included impacts from the following: the COVID-19 pandemic during the study period; tight eligibility criteria, such as excluding patients with (moderate-to-severe) infection and those using non-removable offloading devices; no access to the required technical equipment when ready to recruit due to competing planning issues in clinics; and patients’ ulcer having healed before their first study appointment (Figure 1). 

### 3.2. Participant Characteristics and Primary Outcomes

Table 2 shows that participant characteristics were not statistically significantly different between the studies, including demographic, ulcer, and offloading device characteristics (all *p* > 0.05), except for retinopathy (AU-study: 15% vs. NL-study: 70%, *p* = 0.009).

Overall, 12 (50%) of the 24 participants’ ulcers (missing data *n* = 1) healed at 12 weeks. In the AU-study, 7 (54%) of the 13 participants’ ulcers healed, with the only difference in baseline characteristics being more females in the healed group (Healed: 57% vs. Non-healed: 0%, *p* = 0.026, Table 2). In the NL-study, 5 (45%) of the 11 participants (missing data *n* = 1) healed, with the only differences being shorter diabetes duration (Healed: 8 vs. Non-healed: 26 years, *p* = 0.004) and fewer amputations (Healed: 0% vs. Non-healed: 100%, *p* < 0.001) in the healed group. In the AU-study, three (33%) of the nine participants (missing data *n* = 4) had ≥75% ulcer surface area reduction at 4 weeks. In the NL-study, 5 (45%) of the 11 participants (missing data *n* = 1) had ≥75% ulcer surface area reduction at 4 weeks.

### 3.3. Outcomes of Interest

Table 3 displays all outcomes of interest findings for both studies. In the AU-study, participants who healed had a lower CPTS than those who did not heal in both CPTS models (median CPTS according to model 1: 369 vs. 526 MPa·s/day, and according to model 2: 457 vs. 679 MPa·s/day), although neither difference was statistically significant (both r = 0.26 and *p* = 0.800). In the NL-study, CPTS could only be determined for one participant (who did not heal), as that was the only participant for whom all underlying factors were available. CPTS was 8.7 MPa·s/day, but an association with healing could not be investigated. 

In the AU-study, of the underlying factors that determine CPTS (available in up to 12 participants), we found, descriptively, that participants who healed had slightly higher PPPs (r = 0.19) and largely higher PTIs (r = 0.51) in their offloading devices, slightly lower PPPs (r = 0.22) and largely lower PTIs (r = 0.61) in their regular footwear, a slightly lower daily number of steps (r = 0.16), slightly higher adherence to using their offloading devices (r = 0.26), and a thermal stress response that was slightly higher in their offloading device (r = 0.17) and moderately lower in their regular footwear (r = 0.44) compared to those who did not heal; although, again, no difference found reached statistical significance. In the NL-study, of the underlying factors (available in up to seven participants), those who healed had moderately higher barefoot PPPs and PTIs (both r = 0.40), a slightly higher daily number of steps (r = 0.13), and no difference in adherence (r = 0.00) compared to those who did not heal; although, again, no difference found reached statistical significance (Table 3).

### 3.4. Feasibility Outcomes

Figure 1 shows the data completion for CPTS and all underlying factors. In the AU-study, CPTS data were completed for five (42%) participants at baseline and one (8%) participant at week 8. In the NL-study, CPTS data were completed for one (8%) participant at baseline and zero (0%) participants at week 8. 

In the AU-study, of the underlying factors at baseline, plantar pressure data were completed for 12 (92%) and 11 (85%) in-device and non-device condition participants, respectively, thermal stress response data were completed for 11 (85%) participants in both conditions, activity data were completed for 9 (69%) participants, and adherence data were completed for 5 (42%) participants. At week 8, plantar pressure data were completed for two (15%) participants, thermal stress response data were completed for three (23%) participants, activity data were completed for five (15%) participants, and adherence data were completed for one (8%) participant. In the NL-study, of the underlying factors at baseline (or at week 2 for plantar pressures), plantar pressure data were completed for three (25%) participants in the device and seven (58%) participants barefoot, activity data were completed for seven (58%) participants, and adherence data were completed for four (33%) participants. At week 2, the NL-study had one (8%) drop-out. At week 8, activity data was completed for one (8%) participant and adherence data for zero (0%) participants. 

**Table 3:** Cumulative plantar tissue stress (CPTS) and its underlying factors at the ulcer site.

Participants	Australian Study (*n* = 13)	Healed Ulcer (*n* = 7)	Non-healed Ulcer (*n* = 6)	Effect Size	Netherlands Study (*n* = 12)	Healed Ulcer (*n* = 5) ^a^	Non-healed Ulcer (*n* = 6) ^a^	Effect Size
**CPTS (MPa·s/day)**								
-Model	369.0 [95.8; 514.1] ^(*n* = 5)^	369.0 [112.2; 373.2] ^(*n* = 3)^	525.6 [118.8; 932.4] ^(*n* = 2)^	0.26	8.7 ^(*n* = 1)^	NA	8.7 ^(*n* = 1)^	NA
-Model weighted for thermal stress response	457.4 [24.3; 944.2] ^(*n* = 5)^	457.4 [117.0; 726.7] ^(*n* = 3)^	679.2 [311.8; 1327.2] ^(*n* = 2)^	0.26	NA	NA	NA	NA
**In-shoe plantar pressure**								
-PTI in device (kPa·s)	66.2 [60.0; 74.7] ^(*n* = 12)^	74.7 [66.2; 85.9] ^(*n* = 6)^	60.4 [59.6; 66.2]	0.51	64.7 [48.5, 111.0] ^(*n* = 3)^	NA	64.7 [48.5; 111.0] ^(*n* = 3)^	NA
-PPP in device (kPa)	140.6 [110.9; 302.7] ^(*n* = 12)^	160.8 [105.4; 287.1] ^(*n* = 6)^	140.6 [124.1; 351.3]	0.19	119.4 [81.8, 179.8] ^(*n* = 3)^	NA	119.4 [81.8; 180.0-] ^(*n* = 3)^	NA
-PTI in footwear (kPa·s)	72.2 [67.2; 86.3] ^(*n* = 11)^	87.4 [78.9; 91.6] ^(*n* = 5)^	70.4 [50.5; 72.2]	0.61	NA	NA	NA	NA
-PPP in footwear (kPa)	178.7 [141.4; 236.4] ^(*n* = 11)^	172.4 [134.4; 233.6] ^(*n* = 5)^	189.4 [152.4; 243.8]	0.22	NA	NA	NA	NA
**Barefoot plantar pressure**								
-PTI barefoot (kPa·s)	NA	NA	NA	NA	495.2 [235.7; 626.1] ^(*n* = 7)^	620.1 [327.4; 1062.6] ^(*n* = 4)^	325.4 [235.7; 452.8] ^(*n* = 3)^	0.40
-PPP barefoot (kPa)	NA	NA	NA	NA	646.3 [446.0; 923.8] ^(*n* = 7)^	836.3 [409.4; 1062.6] ^(*n* = 4)^	625.0 [446.0; 641.0] ^(*n* = 3)^	0.40
**Weight-bearing activity (per day)**								
-Number of steps	7444 [1015; 12,456] ^(*n* = 9)^	7444 [988; 10,636] ^(*n* = 5)^	7854 [2197; 25,215] ^(*n* = 4)^	0.16	2146 [1400; 4232] ^(*n* = 7)^	3075 [1600; 5880] ^(*n* = 4)^	2146 [566; 3732] ^(*n* = 3)^	0.13
**Adherence to using device (%)**								
-Strides in device	50.9 [26.6; 68.7] ^(*n* = 5)^	50.9 [37.5; 74.9] ^(*n* = 3)^	35.7 [7.5; 63.9] ^(*n* = 2)^	0.26	48.1 [16.8; 85.5] ^(*n* = 4)^	54.2 [15.9; 92.4] ^(*n* = 2)^	48.1 [17.7, 78.5] ^(*n* = 2)^	0.00
**Thermal stress response**								
-In-device	0.66 [0.30; 1.07] ^(*n* = 11)^	1.35 [−0.19; 3.37] ^(*n* = 6)^	0.42 [0.14; 0.71] ^(*n* = 5)^	0.17	NA	NA	NA	NA
-Non-device	1.45 [−0.15; 2.54] ^(*n* = 11)^	0.98 [0.66; 1.28] ^(*n* = 6)^	1.45 [−0.22; 1.84] ^(*n* = 5)^	0.44	NA	NA	NA	NA

Note: Continuous data are medians [IQ 25th percentile; 75th percentile]. Significant differences are depicted as *** = *p* < 0.001, ** = *p* < 0.01, and * = *p* < 0.05. ^a^ = one primary outcome (i.e., healing at 12 weeks) is missing. If data were missing for a variable, the number of available data are shown in superscript. CPTS = cumulative plantar tissue stress, PTI = pressure–time integral, PPP = peak pressure, and NA = not applicable.

The reasons for not completing the data are shown in Figure 1. At baseline, the reasons mainly included technical issues (AU-study: 31% and NL-study: 50%), followed by non-adherent participants (AU-study: 15% and NL-study: 8%), or a combination of technical issues and non-adherent participants (AU-study: 15% and NL-study: 33%). At follow-up, the main reason was refusal of participants at 4 weeks (AU-study: 31% and NL-study: 33%) and 8 weeks (AU-study: 62% and NL-study: 25%).

Table 4 displays the baseline characteristics of the AU participants for those with and without CPTS data available. In the AU-study, 5 (38%) of the 13 participants had CPTS data available, with the only difference in baseline characteristics being a lower BMI in those with CPTS data available (26 vs. 31 kg/m^2^, *p* = 0.006). In the NL-study, only one participant had CPTS data available; therefore, this analysis was not performed for those participants.

## 4. Discussion

Our aims were to investigate the association between CPTS and ulcer healing in people with diabetes-related plantar foot ulcers using removable offloading treatments and to explore the feasibility of measuring CPTS in this population. We were only able to recruit 25 (25%) participants of the required 100 participants in two observational cohort studies. At 12 weeks, 50% of participants healed from their ulcer. In both studies, the completion of all measures needed to determine CPTS was low, as CPTS could only be determined for five (38%) AU participants and one (8%) NL participant at baseline, and this number declined to one AU participant and zero NL participants during follow-up. The reasons for low completion primarily included the following: (i) technical issues with (sensor) equipment; (ii) non-adherence to use of the weight-bearing activity measurement equipment; and (iii) refusal by patients to undertake follow-up visits. The underpowered findings from the five AU participants showed that CPTS was non-significantly lower in people who healed compared to those who did not heal. In this discussion, we reflect on these underpowered findings and the challenges of measuring CPTS in this population, aiming to advance research in this field by sharing the numerous valuable lessons learned.

### 4.1. The Association between CPTS and Ulcer Healing

CPTS reflects the interplay of multiple factors thought to influence the overall mechanical stresses on a foot ulcer site, including plantar pressure, shear stress, weight-bearing activity, and adherence to using offloading devices or footwear. In the AU-study, CPTS was non-significantly slightly lower in people who healed compared to people who did not heal at 12 weeks (369 vs. 526 MPa·s/day, or (when weighted for shear stress) 457 vs. 679 MPa·s/day). The direction of results was in line with the exploratory study of van Netten et al. [18] (healed: 155 vs. non-healed: 207 MPa·s/day), but that study used a different CPTS model as it did not have quantifiable data on all underlying factors. In the NL-study, CPTS was very low (9 MPa·s/day) in the only participant in whom CPTS could be determined, due to the extremely low weight-bearing activity of this participant (40 daily steps). For the underlying factors, we found no statistically significant differences between people who healed and those who did not in both studies. Regardless, overall, both studies were underpowered due to the low number of participants we were able to recruit and the low number of participants in whom we could successfully complete all measures to determine CPTS. Thus, these findings are better viewed as those of a second pilot study on CPTS in foot ulcer healing. Larger studies remain needed to investigate the value of CPTS, and the multiple underlying individual factors, and its association with ulcer healing. 

### 4.2. Feasability of Measuring CPTS

Our findings on feasibility showed that measuring CPTS is challenging, reflecting that feasibility is currently poor in this population. In both studies, we encountered the issue of incomplete data on CPTS at baseline, and this incompleteness became worse during follow-up. Although measurement time was not formally included as a feasibility measure, we estimated anecdotally that researchers spent approximately 2 h to measure all the underlying factors to determine CPTS. Patients with a foot ulcer typically need multiple time-consuming multidisciplinary treatments each week [6,7]; therefore, this additional CPTS measurement time may have been too much. This is indicated in many potentially eligible patients who initially declined to participate in this study and in many participants who refused (25–62%) to attend follow-up measurements. Our feasibility findings seem to contradict those from the earlier study [18], where CPTS data were completely available for all participants. However, that study used only two measurements (daily steps and in-device plantar pressures), which were measured only once (at baseline). Additionally, adherence was measured via self-report, which has since been found to be unreliable [36], and barefoot pressures and shear stress were not measured. To improve reliability in this study, we used four sensor devices to measure and determine CPTS in our current studies, and we attempted to collect the data three times during follow-up to investigate changes over time. These follow-up measurements of CPTS are important given, for example, the changes in adherence found in other studies to wearing offloading devices over the course of offloading treatment [39]. In the Netherlands, we conducted a similar study also during COVID-19 in people with diabetes who were at risk of developing a foot ulcer, but did not have one [40]. The methods in both studies in the Netherlands were similar, with the exception of different follow-up intervals: follow-ups every 3 months for 1 year instead of every 4 weeks for 12 weeks. This study successfully recruited the number of powered participants and completed data collection on cumulative plantar tissue stress for 52 out of 60 participants at baseline. With no difference in methods at baseline, this suggests that conducting these measurements in people with diabetes but without a foot ulcer is more feasible. We hypothesise that this may be due to people with diabetes-related foot ulcers having poorer health-related quality of life compared to those who healed from such ulcers [41], as well as the more intensive and frequent multidisciplinary clinical treatments they undergo. We suggest that these factors, including poor quality of life, high burden of clinical care, and more frequent research measurements, contributed to the differences in participation and refusal between the two studies. 

At baseline, the main reasons for incomplete CPTS data were technical issues with (sensor) equipment, participant non-adherence to using the weight-bearing activity measurement equipment, and a combination of those reasons. The activity monitor used had to be worn in a belt fitted around people’s waist, which differed from the ankle-worn device used in the earlier study. This belt caused inconveniences for various participants, especially for those with higher BMI, which was shown by their incomplete CPTS measurement data. Adherence to, for example, a wristwatch type of activity sensor may have been better in term of practicality; however, some of these sensors are less accurate [28]. The question whether a less accurate sensor with potentially higher adherence is a better option to measure weight-bearing activity is an important one for future research. Further, measuring adherence using temperature sensors in offloading devices also posed challenges. These challenges included the limited data storage capacity of the sensors, instances where the offloading device changed for clinical reasons in the absence of researchers in the clinic, and situations where patients threw away their offloading device (with temperature sensor) as soon as their ulcer healed, rather than returning them to the clinic. As a result, sensors disappeared, and data readings could not take place in time by the researcher. Finally, the laptop used in the NL-study had an unexpected issue, causing some plantar pressure measurements to be lost before a back-up was made. While this was a very specific issue in this study, this stresses the importance of backing-up data. Furthermore, it stresses the fragility of using different software systems and the ever-increasing challenges in using cybersecurity measures that may block adequate functioning of unique device-specific software. Therefore, all these practical and technical challenges should be considered when designing future CPTS studies in this population.

### 4.3. Strengths and Limitations

A strength of both studies was that these were the first studies designed to prospectively investigate the association between CPTS and its underlying factors with diabetes-related plantar foot ulcer healing in people treated with a removable offloading device. Both studies used validated, objective, and quantifiable methods for all measured underlying factors of CPTS. In addition to the power and feasibility limitations already discussed, we again note the limitation that we were underpowered, with only being able to recruit 25 out of the 100 required participants. We are also limited in not having recorded the exact numbers and reasons for eligible patients who declined to participate in this study, yet the key anecdotal reasons for declining from eligible patients was their reluctance to participate in the multiple follow-ups required to measure CPTS. This seems especially relevant for people with a foot ulcer as CPTS has also been investigated in people without a foot ulcer, where recruitment and data completion was not a problem [40,42]. People without a foot ulcer have limited clinical appointments. This reduces the burden of the extra research-related visits, and may even render these visits a bonus, as they also entail extra foot checks. This is a stark contrast with the clinical burden of people with a foot ulcer. These differences should be taken into account when setting up further research for these populations. In addition, we were challenged by the impact of COVID-19 and related restrictions. Research activities were terminated for multiple months during the COVID-19 pandemic. However, funding for research staff and compensations were not sufficient to continue with the studies, which forced us to terminate the studies before the number of powered participants was reached. During the months that we could recruit participants, tight eligibility criteria and equipment unavailability also hampered adequate participant inflow. Tight eligibility criteria mainly involved excluding patients with moderate-to-severe infection and those using non-removable offloading devices. Up to 50% of people with a foot ulcer develop an infection [23,43], showing that infections are common in this population. However, we had to exclude them because their primary clinical focus is on treating the infection [23]. We excluded non-removable offloading devices because plantar pressure and shear stress measurements cannot be performed in these devices, and these have already been proven as the gold standard treatment [19]. Equipment unavailability concerned the challenge of sharing one Pedar system between research and clinical practice, particularly in the AU-study. This is not unusual: the system is expensive, and for optimal use, it needs to be applied in both settings. However, this requires careful logistic scheduling when (as in our situation) clinics are in different geographical locations. The stress of this scheduling was shown in the errors made, rendering the system, or even patients, unavailable when needed. Because the window for participation in people with a plantar foot ulcer is relatively short due to the primary clinical aim of healing, this also meant some participants were not able to be recruited or followed-up in time, resulting in missing data that cannot be compensated at later time points. 

A limitation not related to feasibility was that we based CPTS only on the number of strides, excluding other weight-bearing activities. However, an earlier study showed that standing also significantly affects CPTS [40]; this is supported by the finding that people with diabetes and a plantar foot ulcer stand almost thrice as long as they walk [39]. Because we did not measure plantar pressure during standing or any other weight-bearing activity, we could not determine CPTS with a model that included more activities [40]. This should be considered in future research, even though it does require even more measurements, prolonging the required participation time and associated burdens. In addition, the results of our study may not be generalizable for weight-bearing activity when considering earlier studies. Contrary to the findings from a recent systematic review, we found that people with foot ulcers in a warmer climate (Brisbane, AU) were more active than the people in a moderate climate (NL) [44]. Thus, these participants may not be representative for the larger population; however, variation in weight-bearing activity was large in the systematic review [44]. Further, we had no insight into the tissue properties of ulcers or surrounding skin from our participants. The physical stress theory of Kluding et al. [45] suggests that the stress on the foot can either be too high or too low, and both may lead to poor ulcer outcomes. Moreover, these stress thresholds may differ per individual [45], suggesting that some patients with high tissue stress may heal, while others with low tissue stress may not heal from their ulcer. As a result, skin and tissue quality at the ulcer site likely also affect the association between CPTS and clinical outcomes. Last, the majority of offloading devices used in this study were custom-made insoles or footwear, while the IWGDF guidelines on offloading foot ulcers recommend the use of knee- or ankle-high devices [19]. However, this aligns with large surveys of clinical practice where the prescription of removable offloading devices is still relatively low [46,47].

### 4.4. Future Directions and Implications

CPTS has been described as a critical concept in the healing and prevention of diabetes-related foot ulcers [9] and is recommended in international guidelines as a potential concept to facilitate clinical decision making in the future [19]. However, since the introduction of this promising concept 20 years ago [17], the observational cohort studies described in this manuscript were the first two studies primarily designed to investigate CPTS in people with a diabetes-related plantar foot ulcer who had all factors underlying CPTS measured objectively. The process to determine cumulative plantar tissue stress is very sensor- and technically driven at this point and hence complex. Despite widespread support for integrating sensors to measure and intervene to improve healing of diabetes-related foot ulcers [48,49,50], our research currently does not support this idea due to low feasibility. We do not recommend taking time-consuming measurements several times within 3 months in people with a diabetes-related foot ulcer. Furthermore, if possible, measurements could be integrated into daily clinical care, taking into account the quality of life of these patients. Ideally, CPTS would be measured in future studies with one sensor device to advance the science and technology of sensor application in people with diabetes-related foot disease. Such a sensor device should measure all the mechanical stresses (i.e., plantar pressure and shear stress) at the foot site of interest and could perhaps even be (temporarily) attached to the foot to measure mechanical stress both while wearing a device and while walking barefoot. Additionally, the sensor should be small enough so that it could be integrated in something that a patient wears (almost) continuously, like within their footwear, their insole, their boot, or their sock. Furthermore, the sensor should incorporate a built-in memory capable of storing data over multiple days. Moreover, the sensor could provide valuable insights into CPTS, plantar pressure, shear stress, level of activity, and adherence of the patient, accessible through a smartphone application. Such a sensor device also gives the advantage of continuous measurement of CPTS, which would enhance generalizability and reduce the number of study visits. Additionally, the sensor device should be easy to use for patients, clinicians, and researchers. Until such a sensor device is available, researchers should use the lessons learned in this study to better balance minimization of participant burdens with maximization of data validity.

Despite the challenges encountered in our study, current exploratory findings from this study and from the earlier pilot study [18] seem to suggest that CPTS is lower in people whose plantar foot ulcer healed when treated with a removable offloading device compared to those who did not heal. We therefore suggest that it remains important to continue investigations in CPTS and its association with healing outcomes in people treated with removable offloading devices because plantar pressure measurements alone do not sufficiently elucidate the underlying mechanisms of ulcer healing in this population [14]. If objective thresholds for ulcer healing can be determined based on CPTS or any of its underlying factors, this could be useful in clinical decision making. Additionally, this may potentially lead to being able to develop new treatments for a CPTS target or facilitate smart devices that could warn people to take preventative action when thresholds are reached [51]. 

## 5. Conclusions

We found that the feasibility of measuring CPTS is currently low in people with a diabetes-related plantar foot ulcer using removable offloading treatment. This is primarily the result of the challenges of having to use multiple sensor devices, with multiple and long measurements, to measure CPTS in this population. The burden of being treated for a diabetes-related foot ulcer while simultaneously participating in research was reflected in a high refusal rate to participate in study measurements. The value of CPTS, and its association with diabetes-related ulcer healing, remains to be investigated in future larger studies taking into account the feasibility findings from the two studies reported here.

## Figures and Tables

**Table 1:** Overview of the outcomes of interest measurements.

Measurements	0 Weeks	2 Weeks	4 Weeks	8 Weeks	12 Weeks
Plantar pressure	AU	NL	AU	AU	
Plantar shear stress	AU		AU	AU	
Daily weight-bearing activity	AU, NL		AU, NL	AU, NL	
Wearing time of offloading device	AU, NL	------------------------------------------------------------->			
Adherence to offloading device	AU, NL		AU, NL	AU, NL	

Note: AU = Australian study and NL = Netherlands study.

**Table 2:** Baseline characteristics.

Participants	Australian Study (*n* = 13)	Healed Ulcer at 12 Weeks (*n* = 7)	Non-Healed Ulcer at 12 Weeks (*n* = 6)	Netherlands Study (*n* = 12)	Healed Ulcer at 12 Weeks (*n* = 5) ^a^	Non-Healed Ulcer at 12 Weeks (*n* = 6) ^a^
**Age (years)**	60.0 [49.0; 72.5]	64.0 [52.0; 75.0]	54.0 [45.3; 66.5]	66.0 [61.3.; 75.0]	67.0 [61.5; 71.5]	63.5 [60.5; 75.5]
**Sex (% (*n*))**		*	*			
-Female	31 (4)	57 (4)	0 (0)	17 (2)	0 (0)	17 (1)
-Male	69 (9)	43 (3)	100 (6)	83 (10)	100 (5)	83 (5)
**Body mass index (kg/m^2^)**	28.4 [26.2; 31.9]	27.5 [26.0; 30.3]	30.8 [27.8; 41.3]	34.0 [26.5; 36.2]	34.6 [34.0; 37.1]	29.9 [26.0; 37.7]
**Diabetes type (% (*n*))**						
-Type 1	0 (0)	0 (0)	0 (0)	17 (2)	0 (0)	33 (2)
-Type 2	100 (13)	100 (7)	100 (6)	83 (10)	100 (5)	67 (4)
**Diabetes duration (years)**	15.0 [7.0; 20.0] ^(*n* = 11)^	17.0 [10.0; 21.8] ^(*n* = 6)^	7.0 [7.0; 20.0] ^(*n* = 5)^	15.5 [8.0; 29.0]	8.0 [5.3; 11.5] **	26.0 [19.0; 41.0] **
**HbA1c (mmol/mol)**	7.4 [6.3; 9.0] ^(*n* = 9)^	7.5 [6.5; 8.7] ^(*n = 4*)^	7.4 [6.1; 9.4] ^(*n* = 5)^	7.3 [6.7; 8.5] ^(*n* = 10)^	7.3 [6.6; 7.9]	8.3 [7.2; 10.4] ^(*n* = 4)^
**Ulcer history (% (*n*))**						
-Yes	92 (12)	86 (6)	100 (6)	83 (10)	80 (4)	100 (6)
-No	8 (1)	14 (1)	0 (0)	17 (2)	20 (1)	0 (0)
**Amputation history (% (*n*))**					***	***
-Yes	46 (6)	29 (2)	67 (4)	50 (6)	0 (0)	100 (6)
-No	54 (7)	71 (5)	33 (2)	50 (6)	100 (5)	0 (0)
**Peripheral artery disease (% (*n*))**				^(*n* = 11)^		^(*n* = 5)^
-Yes	8 (1)	14 (1)	0 (0)	18 (2)	0 (0)	40 (2)
-No	92 (12)	86 (6)	100 (6)	82 (9)	100 (5)	60 (3)
**Nephropathy (% (*n*))**				^(*n* = 11)^		^(*n* = 5)^
-Yes	15 (2)	14 (1)	17 (1)	18 (2)	20 (1)	20 (1)
-No	85 (11)	86 (6)	83 (5)	82 (9)	80 (4)	80 (4)
**Retinopathy (% (*n*))**	**			** ^(*n* = 10)^	^(*n* = 4)^	^(*n* = 5)^
-Yes	15 (2)	29 (2)	0 (0)	70 (7)	100 (4)	60 (3)
-No	85 (11)	71 (5)	100 (6)	30 (3)	0 (0)	40 (2)
**Ulcer duration before study (weeks)**	6.0 [3.0; 24.0] ^(*n* = 12)^	6.5 [1.8; 72.0] ^(*n* = 6)^	6.0 [3.8; 29.0]	2.5 [2.0; 5.8]	2.0 [1.0; 12.5]	2.5 [1.5; 4.3]
**Ulcer site (% (*n*))**						
-Hallux	23 (3)	14 (1)	33 (2)	33 (4)	80 (4)	17 (1)
-Forefoot	62 (8)	71 (5)	50 (3)	50 (6)	20 (1)	50 (3)
-Midfoot	0 (0)	0 (0)	0 (0)	17 (2)	0 (0)	33 (2)
-Rearfoot	15 (2)	14 (1)	17 (1)	0 (0)	0 (0)	0 (0)
**Ulcer size (mm^2^)**	12.0 [5.5; 77.0]	9.0 [4.0; 20.0]	23.0 [7.0; 62.0]	20.5 [4.9; 177.5]	16.0 [4.1; 37.5]	134.0 [5.9; 550.0]
**Ulcer depth (mm)**	1.0 [1.0; 2.5]	1.0 [1.0; 3.0]	1.0 [1.0; 2.0]	1.0 [1.0; 2.0] ^(*n* = 9)^	1.0 [1.0; 1.8] ^(*n* = 4)^	2.0 [1.3; 2.0] ^(*n* = 4)^
**Texas classification (% (*n*))**				^(*n* = 11)^		^(*n* = 5)^
-Superficial (1)	100 (13)	100 (7)	100 (6)	82 (9)	92 (11)	80 (4)
-Penetrate to tendon or capsule (2)	0 (0)	0 (0)	0 (0)	18 (2)	8 (1)	20 (1)
-Penetrate to bone (3)	0 (0)	0 (0)	0 (0)	0 (0)	0 (0)	0 (0)
**Mild infection (% (*n*))**				^(*n* = 11)^		^(*n* = 5)^
-Yes	0 (0)	0 (0)	0 (0)	18 (2)	20 (1)	20 (1)
-No	100 (13)	100 (7)	100 (6)	82 (9)	80 (4)	80 (4)
**Ischemia (% (*n*))**				^(*n* = 11)^		^(*n* = 5)^
-Yes	8 (1)	14 (1)	0 (0)	0 (0)	0 (0)	0 (0)
-No	92 (12)	86 (6)	100 (6)	100 (11)	100 (5)	100 (5)
**Offloading device used (% (*n*))**						
-Removable knee-high	15 (2)	29 (2)	0 (0)	8 (1)	0 (0)	17 (1)
-Removable ankle-high	23 (3)	14 (1)	33 (2)	17 (2)	20 (1)	17 (1)
-Custom-made insoles or footwear	62 (8)	57 (4)	67 (4)	75 (9)	80 (4)	67 (4)

Note: Continuous data are medians [IQ 25th percentile; 75th percentile] and discrete data are percentages (number of). ^a^ = one primary outcome (i.e., healing at 12 weeks) is missing. Significant differences are depicted as *** = *p* < 0.001, ** = *p* < 0.01, and * = *p* < 0.05. If data were missing for a variable, the number of available data are shown in superscript.

**Table 4:** Baseline characteristics compared between participants for whom we had and did not have CPTS data available.

Participants	CPTS Available (*n* = 5)	CPTS Not Available (*n* = 8)	*p*-Value
**Age (years)**	56.0 [47.5; 75.5]	61.5 [47.5; 68.5]	1.000
**Sex (% (*n*))**			0.569
-Female	40 (2)	25 (2)	
-Male	60 (3)	75 (6)	
**BMI (kg/m^2^)**	26.2 [23.3; 27.4]	30.9 [28.6; 36.7]	0.006 **
**Diabetes type (% (*n*))**			NA
-Type 1	0 (0)	0 (0)	
-Type 2	100 (5)	100 (8)	
**Diabetes duration (years)**	17.5 [9.0; 25.3] ^(*n* = 4)^	13.0 [7.0; 19.0] ^(*n* = 7)^	0.315
**HbA1c (mmol/mol)**	6.6 [6.1; 8.4] ^(*n =* 4)^	7.9 [6.9; 9.4] ^(*n* = 5)^	0.190
**Ulcer history (% (*n*))**			0.188
-Yes	80 (4)	100 (8)	
-No	20 (1)	0 (0)	
**Amputation history (% (*n*))**			0.135
-Yes	20 (1)	63 (5)	
-No	80 (4)	38 (3)	
**Peripheral artery disease (% (*n*))**			0.188
-Yes	20 (1)	0 (0)	
-No	80 (4)	100 (8)	
**Nephropathy (% (*n*))**			0.052
-Yes	40 (2)	0 (0)	
-No	60 (3)	100 (8)	
**Retinopathy (% (*n*))**			0.052
-Yes	40 (2)	0 (0)	
-No	60 (3)	100 (8)	
**Ulcer duration before study (weeks)**	24.0 [5.5; 185.0] ^(*n* = 4)^	3.5 [2.3; 15.5]	0.073
**Ulcer site (% (*n*))**			0.231
-Hallux	0 (0)	38 (3)	
-Forefoot	80 (4)	50 (4)	
-Midfoot	0 (0)	0 (0)	
-Rearfoot	20 (1)	13 (1)	
**Ulcer size (mm^2^)**	7.0 [3.0; 20.0]	19.0 [8.3; 148.5]	0.127
**Ulcer depth (mm)**	2.0 [1.0; 5.0]	1.0 [1.0; 1.0]	0.171
**Texas classification (% (*n*))**			NA
-Superficial (1)	100 (5)	100 (8)	
-Penetrate to tendon or capsule (2)	0 (0)	0 (0)	
-Penetrate to bone (3)	0 (0)	0 (0)	
**Infection (% (*n*))**			NA
-Yes	0 (0)	0 (0)	
-No	100 (5)	100 (8)	
**Ischemia (% (*n*))**			0.188
-Yes	20 (1)	0 (0)	
-No	80 (4)	100 (8)	
**Offloading device used (% (*n*))**			0.296
-Removable knee-high	20 (1)	13 (1)	
-Removable ankle-high	0 (0)	38 (3)	
-Custom-made insoles or footwear	80 (4)	50 (4)	

Note: Continuous data are medians [IQ 25th percentile; 75th percentile] and discrete data are percentages (number of). Significant differences are depicted as *** = *p* < 0.001, ** = *p* < 0.01, and * = *p* < 0.05. If data were missing for a variable, the number of available data are shown in superscript. NA = not applicable.

## Data Availability

The data presented in this study are available on request from the corresponding author.

## References

[1] GBD 2019 Diseases and Injuries Collaborators (2020). Global burden of 369 diseases and injuries in 204 countries and territories, 1990–2019: A systematic analysis for the Global Burden of Disease Study 2019. Lancet.

[2] International Diabetes Federation (2021). IDF Diabetes Atlas, 10th ed. https://www.diabetesatlas.org.

[3] Cho N.H., Shaw J.E., Karuranga S., Huang Y., da Rocha Fernandes J.D., Ohlrogge A.W., Malanda B. (2018). IDF Diabetes Atlas: Global estimates of diabetes prevalence for 2017 and projections for 2045. Diabetes Res. Clin. Pract..

[4] Zhang Y., Lazzarini P.A., McPhail S.M., van Netten J.J., Armstrong D.G., Pacella R.E. (2020). Global disability burdens of diabetes-related lower-extremity complications in 1990 and 2016. Diabetes Care.

[5] Lazzarini P.A., Raspovic K.M., Meloni M., van Netten J.J. (2023). A new declaration for feet’s sake: Halving the global diabetic foot disease burden from 2% to 1% with next generation care. Diabetes Metab. Res. Rev..

[6] Schaper N.C., Van Netten J.J., Apelqvist J., Bus S.A., Fitridge R., Game F., Monteiro-Soares M., Senneville E., on behalf of the I.E. (2023). Practical guidelines on the prevention and management of diabetes-related foot disease (IWGDF 2023 update). Diabetes Metab. Res. Rev..

[7] Vileikyte L., Crews R.T., Reeves N.D. (2017). Psychological and Biomechanical Aspects of Patient Adaptation to Diabetic Neuropathy and Foot Ulceration. Curr. Diab. Rep..

[8] Boulton A.J.M., Vileikyte L., Ragnarson-Tennvall G., Apelqvist J. (2005). The global burden of diabetic foot disease. Lancet.

[9] Lazzarini P.A., Crews R.T., van Netten J.J., Bus S.A., Fernando M.E., Chadwick P.J., Najafi B. (2019). Measuring Plantar Tissue Stress in People With Diabetic Peripheral Neuropathy: A Critical Concept in Diabetic Foot Management. J. Diabetes Sci. Technol..

[10] McDermott K., Fang M., Boulton A.J.M., Selvin E., Hicks C.W. (2023). Etiology, Epidemiology, and Disparities in the Burden of Diabetic Foot Ulcers. Diabetes Care.

[11] Monteiro-Soares M., Boyko E.J., Ribeiro J., Ribeiro I., Dinis-Ribeiro M. (2012). Predictive factors for diabetic foot ulceration: A systematic review. Diabetes Metab. Res. Rev..

[12] Crews R.T., King A.L., Yalla S.V., Rosenblatt N.J. (2018). Recent advances and future opportunities to address challenges in offloading diabetic feet: A mini-review. Gerontology.

[13] Wu S.C., Crews R.T., Armstrong D.G. (2005). The pivotal role of offloading in the management of neuropathic foot ulceration. Curr. Diab. Rep..

[14] Lazzarini P.A., Armstrong D.G., Crews R.T., Gooday C., Jarl G., Kirketerp-Moller K., Viswanathan V., Bus S.A. (2023). Effectiveness of offloading interventions for people with diabetes-related foot ulcers: A systematic review and meta-analysis. Diabetes Metab. Res. Rev..

[15] Raspovic A., Landorf K.B. (2014). A survey of offloading practices for diabetes-related plantar neuropathic foot ulcers. J. Foot Ankle Res..

[16] Wu S.C., Jensen J.L., Weber A.K., Robinson D.E., Armstrong D.G. (2008). Use of pressure offloading devices in diabetic foot ulcers do we practice what we preach?. Diabetes Care.

[17] Maluf K.S., Mueller M.J. (2003). Comparison of physical activity and cumulative plantar tissue stress among subjects with and without diabetes mellitus and a history of recurrent plantar ulcers. Clin. Biomech..

[18] van Netten J.J., van Baal J.G., Bril A., Wissink M., Bus S.A. (2018). An exploratory study on differences in cumulative plantar tissue stress between healing and non-healing plantar neuropathic diabetic foot ulcers. Clin. Biomech..

[19] Bus S.A., Armstrong D.G., Crews R.T., Gooday C., Jarl G., Kirketerp-Moller K., Viswanathan V., Lazzarini P.A. (2023). Guidelines on offloading foot ulcers in persons with diabetes (IWGDF 2023 update). Diabetes Metab. Res. Rev..

[20] van Netten J.J., Bus S.A., Apelqvist J., Chen P., Chuter V., Fitridge R., Game F., Hinchliffe R.J., Lazzarini P.A., Mills J. (2023). Definitions and criteria for diabetes-related foot disease (IWGDF 2023 update). Diabetes Metab. Res. Rev..

[21] Jeffcoate W.J., Bus S.A., Game F.L., Hinchliffe R.J., Price P.E., Schaper N.C. (2016). Reporting standards of studies and papers on the prevention and management of foot ulcers in diabetes: Required details and markers of good quality. Lancet Diabetes Endocrinol..

[22] Mills J.L., Conte M.S., Armstrong D.G., Pomposelli F.B., Schanzer A., Sidawy A.N., Andros G. (2014). The society for vascular surgery lower extremity threatened limb classification system: Risk stratification based on Wound, Ischemia, and foot Infection (WIfI). J. Vasc. Surg..

[23] Senneville É., Albalawi Z., van Asten S.A., Abbas Z.G., Allison G., Aragón-Sánchez J., Embil J.M., Lavery L.A., Alhasan M., Oz O. (2023). IWGDF/IDSA guidelines on the diagnosis and treatment of diabetes-related foot infections (IWGDF/IDSA 2023). Diabetes Metab. Res. Rev..

[24] Phelan E.A., Mahoney J.E., Voit J.C., Stevens J.A. (2015). Assessment and Fall Risk in Primary Health care. Physiol. Behav..

[25] Price C., Parker D., Nester C. (2016). Validity and repeatability of three in-shoe pressure measurement systems. Gait Posture.

[26] Arts M.L.J., Bus S.A. (2011). Twelve steps per foot are recommended for valid and reliable in-shoe plantar pressure data in neuropathic diabetic patients wearing custom made footwear. Clin. Biomech..

[27] Bus S.A., Lange A. (2005). De A comparison of the 1-step, 2-step, and 3-step protocols for obtaining barefoot plantar pressure data in the diabetic neuropathic foot. Clin. Biomech..

[28] Rabinovich R.A., Louvaris Z., Raste Y., Langer D., Van Remoortel H., Giavedoni S., Burtin C., Regueiro E.M.G., Vogiatzis I., Hopkinson N.S. (2013). Validity of physical activity monitors during daily life in patients with COPD. Eur. Respir. J..

[29] de Groot S., Nieuwenhuizen M.G. (2013). Validity and reliability of measuring activities, movement intensity and energy expenditure with the DynaPort MoveMonitor. Med. Eng. Phys..

[30] Van Schooten K.S., Rispens S.M., Elders P.J.M., Lips P., Van Dieën J.H., Pijnappels M. (2015). Assessing physical activity in older adults: Required days of trunk accelerometer measurements for reliable estimation. J. Aging Phys. Act..

[31] Matthews C.E., Ainsworth B.E., Thompson R.W., Bassett D.R. (2002). Sources of variance in daily physical activity levels as measured by an accelerometer. Med. Sci. Sports Exerc..

[32] Dijkstra B., Kamsma Y., Zijlstra W. (2010). Detection of gait and posture using a miniaturised triaxial accelerometer-based system: Accuracy in community-dwelling older adults. Age Ageing.

[33] Lutjeboer T., Van Netten J.J., Postema K., Hijmans J.M. (2018). Validity and feasibility of a temperature sensor for measuring use and non-use of orthopaedic footwear. J. Rehabil. Med..

[34] Menz H.B., Bonanno D.R. (2021). Objective measurement of adherence to wearing foot orthoses using an embedded temperature sensor. Med. Eng. Phys..

[35] (2021). Groningen Algorithm, Version 2. https://github.com/CHulshof/Orthotimer_algorithm.

[36] Jarl G., Hulshof C.M., Busch-Westbroek T.E., Bus S.A., van Netten J.J. (2023). Adherence and Wearing Time of Prescribed Footwear among People at Risk of Diabetes-Related Foot Ulcers: Which Measure to Use?. Sensors.

[37] Wrobel J.S., Ammanath P., Le T., Luring C., Wensman J., Grewal G.S., Najafi B., Pop-Busui R. (2014). A novel shear reduction insole effect on the thermal response to walking stress, balance, and gait for diabetic neuropathy. J. Diabetes Sci. Technol..

[38] Cohen J. (2013). Statistical Power Analysis for the Behavioral Sciences.

[39] Najafi B., Grewal G.S., Bharara M., Menzies R., Talal T.K., Armstrong D.G. (2017). Can’t Stand the Pressure: The Association between Unprotected Standing, Walking, and Wound Healing in People with Diabetes. J. Diabetes Sci. Technol..

[40] Hulshof C.M., Van Netten J.J., Oosterhof C.M., Der J.V., Pijnappels M., Bus S.A. (2024). New biomechanical models for cumulative plantar tissue stress assessment in people with diabetes at high risk of foot ulceration. J. Biomech..

[41] Perrin B.M., van Netten J.J., aan de Stegge W.B., Busch-Westbroek T.E., Bus S.A. (2022). Health-related quality of life and associated factors in people with diabetes at high risk of foot ulceration. J. Foot Ankle Res..

[42] Waaijman R., De Haart M., Arts M.L.J., Wever D., Verlouw A.J.W.E., Nollet F., Bus S.A. (2014). Risk factors for plantar foot ulcer recurrence in neuropathic diabetic patients. Diabetes Care.

[43] Cortes-Penfield N.W., Armstrong D.G., Brennan M.B., Fayfman M., Ryder J.H., Tan T.W., Schechter M.C. (2023). Evaluation and Management of Diabetes-related Foot Infections. Clin. Infect. Dis..

[44] Van Netten J.J., Fijen V.M., Bus S.A. (2022). Weight-bearing physical activity in people with diabetes-related foot disease: A systematic review. Diabetes Metab. Syndr. Clin. Res. Rev..

[45] Kluding P.M., Bareiss S.K., Hastings M., Marcus R.L., Sinacore D.R., Mueller M.J. (2017). Physical Training and Activity in People With Diabetic Peripheral Neuropathy: Paradigm Shift. Phys. Ther..

[46] Lazzarini P.A., Jarl G. (2021). Knee-high devices are gold in closing the foot ulcer gap: A review of offloading treatments to heal diabetic foot ulcers. Medicina.

[47] Samuelson K.L., Kiefer C.T., Wu S.C., Crews R.T. (2020). Changing Perspectives: Offloading a Patient With a Diabetic Foot Ulcer as Opposed to Offloading a Diabetic Foot Ulcer. Foot Ankle Spec..

[48] Srass H., Ead J.K., Armstrong D.G. (2023). Adherence and the Diabetic Foot: High Tech Meets High Touch?. Sensors.

[49] Ruder K. (2024). Diabetic Foot Infections and Amputations Are All Too Common—Here’s What Could Move the Needle. J. Am. Med. Assoc..

[50] Bus S.A., Reeves N.D., Armstrong D.G., Najafi B. (2024). Offloading and adherence through technological advancements: Modern approaches for better foot care in diabetes. Diabetes Metab. Res. Rev..

[51] Park C., Mishra R., Vigano D., Macagno M., Rossotti S., D’Huyvetter K., Garcia J., Armstrong D.G., Najafi B. (2022). Smart Offloading Boot System for Remote Patient Monitoring: Toward Adherence Reinforcement and Proper Physical Activity Prescription for Diabetic Foot Ulcer Patients. J. Diabetes Sci. Technol..

